# Growth Failure and Excessive Weight Gain in a 10 Year Old Male With Obesity: Approach to Diagnosis, Management, and Treatment of Acquired Hypothyroidism

**DOI:** 10.3389/fped.2018.00166

**Published:** 2018-06-19

**Authors:** Tiffany Schumaker, Marisa Censani

**Affiliations:** Division of Pediatric Endocrinology, Department of Pediatrics, Weill Cornell Medicine, New York Presbyterian Hospital, New York, NY, United States

**Keywords:** weight gain, obesity, hypothyroidism, Hashimoto's thyroiditis, growth

## Abstract

We report the case of a 10 year old male with severe obesity who presented with a 2 year history of significant growth failure and excessive weight gain that was subsequently diagnosed with serum negative Hashimoto's thyroiditis and acquired hypothyroidism. Initial investigations revealed a markedly elevated thyroid stimulating hormone (TSH) concentration >100 uIU/mL and an undetectable free thyroxine with total thyroxine 0.56 ug/dL. Thyroid antibodies were negative, however ultrasound findings were consistent with Hashimoto's thyroiditis. After treatment with levothyroxine supplementation, he had significant weight loss and marked improvement in his growth velocity. This case emphasizes the need to recognize excessive weight gain and growth failure as an initial presentation of Hashimoto's thyroiditis and highlights management and approach to treatment. Diagnosis and treatment is vital as prolonged undiagnosed hypothyroidism can result in incomplete catch up growth and compromised final adult height.

## Background

Hypothyroidism is the most common disorder of thyroid function in children. Primary hypothyroidism is characterized by high serum thyroid stimulating hormone (TSH) and low serum free thyroxine (T4) concentrations. Autoimmune, or Hashimoto's, thyroiditis is the most common cause of acquired thyroid disease in children and adolescents in the western world. Although autoimmune thyroiditis, is usually characterized by positive serum anti-thyroperoxidase (TPO) antibodies and/or positive thyroglobulin antibodies, about 5% of patients have no measurable thyroid antibodies (1). Hypothyroidism can be associated with fatigue, constipation, dry skin, cold intolerance, coarse, and brittle hair; however, growth delay may present for years before other symptoms occur. Although change in school performance is uncommon at diagnosis and more often declines, it may actually improve in patients with long standing hypothyroidism, and can delay diagnosis secondary to decreased distractibility and improved concentration. Given the long term effects of prolonged undiagnosed hypothyroidism resulting in incomplete catch up growth and compromised final adult height, this case emphasizes the importance of recognizing the combination of excessive weight gain and growth failure, even in the absence of additional symptoms, as an initial presentation of acquired hypothyroidism in a child.

## Case presentation

A 10 year old male was referred to endocrinology clinic for evaluation of obesity, rapid weight gain, and growth deceleration. His mother noted he was previously one of the tallest children in his class, but now was one of the shortest. Review of previous growth charts revealed growth at the 90th percentile for height at 8 years of age with decrease to the 75th percentile at 9 years, and the 50th percentile by 10 years. His weight was consistently at the 95th percentile, but he had gained 5.5 kg (12 lbs) in the past year with weight now at the 97th percentile and body mass index (BMI) 27.5 kg/m2 at the 99th percentile, meeting criteria for extreme obesity.

His mother noted he had been markedly hyperactive as a child and that this behavior had decreased over the past 1–2 years with great improvement in his grades over the past year. His medical history was unremarkable and he did not take medication. Review of systems was unremarkable and he denied fatigue, muscle weakness, constipation, or cold intolerance. He had a good energy level and there were no recent changes in appetite or concentration. He had occasional dry skin. Family history was remarkable for maternal grandmother and mother with hypothyroidism. His midparental target height was 176.5 cm (69.5 inches) at the 50th percentile for height.

On physical examination, the patient measured 134.9 cm (26th percentile) and weighed 50.2 kg (97th percentile) with BMI 27.5 kg/m2 (99th percentile). He had a normal blood pressure 104/55 mm Hg and heart rate of 84 bpm. The patient was well appearing without dysmorphic features and had a normal affect. He had cherubic facies and fundi appeared normal. His thyroid was palpable and smooth with right and left lobe each measuring 4 cm with no lymphadenopathy. His chest, heart, and abdomen were normal. He had Tanner stage 1 genital development with 3 cc testes and no pubic hair. Skin examination was negative for rash, acanthosis, or striae.

## Final diagnosis

Laboratory evaluation revealed a markedly elevated TSH (thyroid stimulating hormone) concentration >100 uIU/mL (0.5–4.3 uIU/mL), an undetectable free T4 (thyroxine) < 0.25 ng/dL (0.9–1.4 ng/dL), and total T4 0.56 ug/dL (5.6–14.9 ug/dL) with negative thyroid peroxidase and antithyroglobulin antibodies. A thyroid ultrasound showed an enlarged heterogeneous thyroid consistent with autoimmune thyroiditis.

## Clinical course

**Figure 1:**
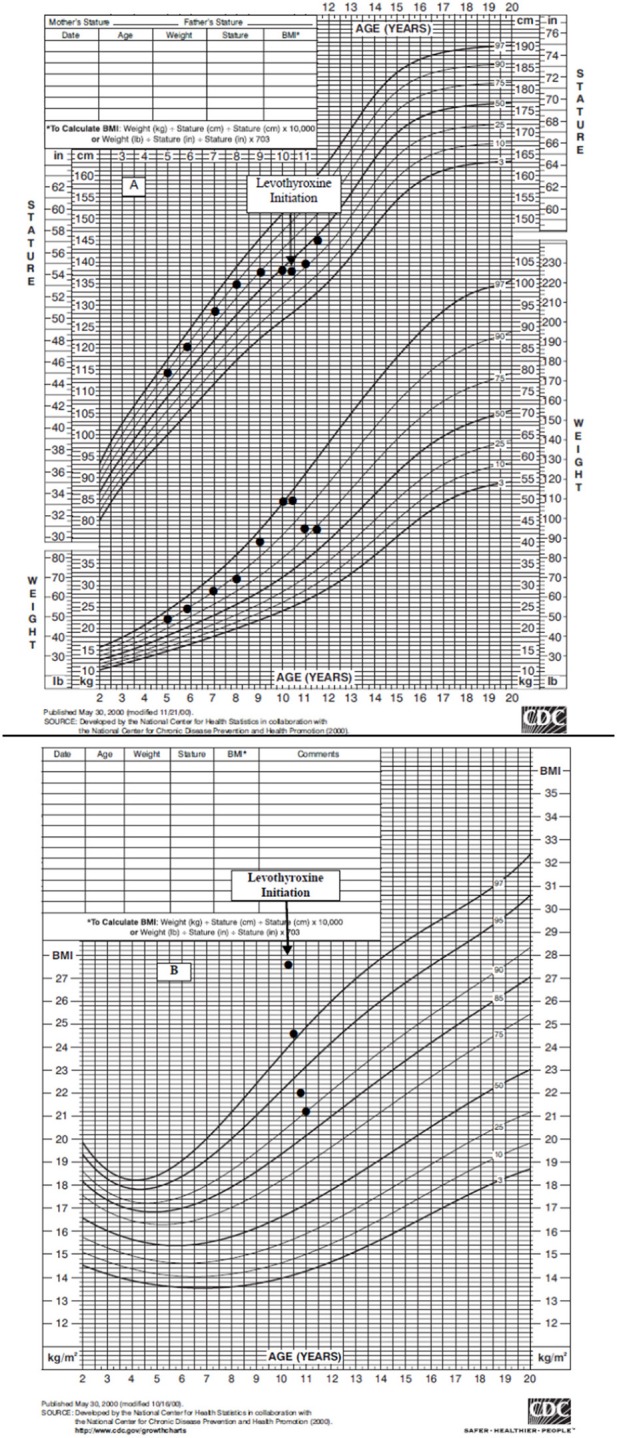
**(A)** Boys Stature-for-age and Weight-for-age Curve. Linear Growth Curve reveals a crossing of percentiles from the 90th to the 25th percentile prior to levothyroxine initiation. Weight curve shows excessive weight gain and subsequent weight loss after levothyroxine initiation. **(B)** Body mass index-for-age Curve. BMI shows a significant decline from severe obesity to overweight status after levothyroxine initiation.

The patient was started on levothyroxine 50 mcg (1 mcg/kg) daily with dose titrated over the next several months (Table 1). Normalization of thyroid levels was achieved on levothyroxine 100 mcg daily with TSH 0.55 uIU/mL, free T4 1.6 ng/dL, and total T4 9.6 ug/dL at 7 months post initiation of levothyroxine supplementation. His growth parameters dramatically improved on treatment (Figure 1). His weight decreased from 50.2 kg (97th percentile) to 43.4 kg (86th percentile) with 6.8 kg (15 lb) weight loss, and BMI decreased from 27.5 kg/m2 (99th percentile) to 22 kg/m2 (93rd percentile). By 7 months post initiation of treatment, his height increased from 134.9 cm (26th percentile) to 140.5 cm (42nd percentile) with 5.6 cm (2.2 inches) of growth in 7 months, giving an annualized growth velocity of 9.7 cm (3.8 inches) per year.

**Table 1:** Thyroid function tests at presentation and months post levothyroxine initiation.

	**Range**	**Presentation**	**1 month**	**3 months**	**5 months**	**7 months**
TSH (uIU/mL)	0.5–4.3	>100	65.45	10.86	4.42	1.82
Free T4 (ng/dL)	0.9–1.4	0.25	0.68	1.1	1.4	1.03
Total T4 (ug/dL)	5.6–14.9	0.56	5.69	7.1	11.2	9.34
TPO-Ab (IU/mL)	0.0–9.0	4.61				
Tg-Ab (IU/mL)	0.0–4.0	1.51				

*TSH, Thyroid stimulating hormone; Free T4, free thyroxine; Total T4, total thyroxine; TPO-Ab, Antithyroid peroxidase antibodies; Tg-Ab, Anti-thyroglobulin antibodies*.

## Discussion

This case highlights that pediatric patients presenting with excessive weight gain and a declining growth velocity with crossing of height percentiles should be evaluated for hypothyroidism, even in the absence of other associated features of hypothyroidism including constipation, declining school performance, or cold intolerance. Acquired hypothyroidism can be related to thyroid disease (primary hypothyroidism) or hypothalamic-pituitary disease (central hypothyroidism). However, most childhood cases of hypothyroidism are primary and are caused by chronic autoimmune thyroiditis. It usually presents in adolescence and has a female predilection, affecting females three to five times more commonly than males (1). Other rare causes of acquired hypothyroidism include excess iodine ingestion, anti-thyroid drugs and thyroid injury secondary to external radiation, thyroidectomy or infiltrative diseases. Iodine deficiency also remains an important cause of acquired hypothyroidism worldwide.

Autoimmune hypothyroidism is characterized by antibodies against thyroglobulin and thyroperoxidase (TPO) and by lymphocytic infiltration of the thyroid gland, which can result in thyromegaly (2). It has been documented that although around 90% of patients have positive anti-TPO antibodies and 50% have positive thyroglobulin antibodies, about 5% of patients with a diagnosis of Hashimoto's thyroiditis based on clinical grounds or by appearance on ultrasound have no measurable thyroid antibodies. In cases of seronegative autoimmune thyroiditis, thyroid ultrasound examination is considered the cornerstone for diagnosis as well as for differentiating this chronic inflammatory condition from other forms of non-autoimmune hypothyroidism of genetic origin (3).

In has been reported in adult literature that patients with Hashimoto's thyroiditis and negative thyroid antibodies have a milder form of the disease; however, pediatric literature regarding the clinical course of seronegative thyroiditis is sparse (3). Our patient presented with significant effects on growth parameters. Hashimoto's disease may be associated with a euthyroid state, hypothyroidism or transient hyperthyroidism. Patients with anti-TPO antibodies are at risk for developing hypothyroidism, however, it is known that some individuals with large goiters and high antibody levels can remain euthyroid for many years (4).

Among children who develop thyroid autoimmunity, a significant number go on to develop hypothyroidism, with one recent study showing that of the children with positive thyroid antibodies, about 20% began treatment with levothyroxine within 3 years after diagnosis. A concurrent diagnosis of celiac disease, an elevated baseline TSH and higher antithyroid antibody titers have been associated with an increased risk of progression to hypothyroidism (5).

## Clinical presentation and natural history

The most common presentation of thyroiditis is thyroid gland enlargement, followed by clinical symptoms of hypothyroidism and then those found incidentally as part of routine screening or a work up for an unrelated condition (1, 6). The most common clinical manifestation of hypothyroidism in children is actually declining growth velocity, which can also be accompanied by increased and sometimes excessive weight gain. The growth delay is usually insidious in onset, and may be present for a few years before other symptoms occur (1). Children that present with severe growth failure usually have very low T4 values, often < 2 ug/dL and an extremely elevated TSH, often >250 uIU/mL. Unfortunately, prolonged juvenile acquired hypothyroidism that is undiagnosed can result in incomplete catch up growth and the severity of the height deficit is proportionate to the duration of the hypothyroidism (7).

The signs and symptoms of hypothyroidism in children are often elusive and depend on severity and duration. Hypothyroidism can be associated with fatigue, constipation, dry skin, cold intolerance, coarse and brittle hair, and myxedema. However, these symptoms may escape detection and may only be discovered retrospectively. Change in school performance is uncommon at diagnosis and was documented in only 3.3% of children in one study (8). Although performance more often declines, it may actually improve in patients with long standing hypothyroidism and can delay diagnosis. This is thought to be secondary to decreased distractibility and improved concentration.

Of note, it is important to differentiate that in individuals with obesity, a slightly elevated TSH level in the setting of a normal T4 are physiologic and reflect the body's attempt to increase metabolism and do not necessitate treatment. With weight loss, TSH levels often normalize in these patients with obesity (9).

## Treatment and prognosis

The treatment of choice for acquired hypothyroidism is daily oral thyroxine. Recommendations for dosing are not standardized as there is variation in the effect of thyroxine replacement in different individuals. The initiation of treatment and subsequent monitoring typically requires the assistance of the pediatric endocrinologist. A low starting dose of 0.25–0.5 mcg/kg/day has been recommended to avoid potential acceleration of skeletal age advancement disproportionate to height gains (2). Once TSH concentrations normalize and catch up growth is complete, thyroid function tests can be monitored throughout puberty 2–3 times yearly (10).

In most patients, hypothyroidism will be permanent. However, there may be periods of remission with thyroid function fluctuation and therefore it may be appropriate to trial patients off thyroxine for 6–8 weeks after the completion of growth and puberty to determine if hypothyroidism is permanent (10). Those that had elevated TSH values while on thyroxine replacement do not need a trial of thyroxine withdrawal.

## Conclusion

Given that prolonged undiagnosed hypothyroidism can result in incomplete catch up growth and a compromised final adult height, thyroid function tests should be strongly considered in the initial evaluation of a child with growth deceleration and weight gain. Our patient's decreasing height percentile and increasing weight, on review of his pediatrician's growth charts, were the major clues to his underlying diagnosis. His improved behavior was subsequently attributed to suppression of his ADHD symptoms due to his severe hypothyroidism and highlights the importance of recognizing change in academic performance as another sign of thyroid dysfunction. In contrast to adult literature with a milder disease state documented in serum negative autoimmune thyroiditis, our patient had severe overt hypothyroidism and significantly affected growth parameters, suggesting a more severe presentation in the pediatric population. Regardless of etiology, it is imperative to recognize growth failure as the possible initial presentation of acquired hypothyroidism in pediatric patients, even in the absence of commonly described symptoms. Laboratory assessment and early intervention can ultimately prevent the development of symptomatology and the loss of final adult height.

## Ethics statement

Written informed consent was obtained from the patient's parent prior to presenting the case.

## Author contributions

TS and MC have directly cared for the patient. TS and MC contributed to the research, writing, and editing of the manuscript, approved the final version, and agreed to be accountable for the content of this work.

### Conflict of interest statement

The authors declare that the research was conducted in the absence of any commercial or financial relationships that could be construed as a potential conflict of interest.
